# Supplementary material for: Ebola virus disease in the Democratic Republic of the Congo, 1976-2014
Source: eLife. 2015 Nov 3;4:e09015. doi: 10.7554/eLife.09015 (PMC4629279; doi:10.7554/eLife.09015)
Supplement: Supplementary file 2. — Repository of the interventions carried out during all outbreaks in the Democratic Republic of the Congo. DOI: http://dx.doi.org/10.7554/eLife.09015.018 [file elife09015s002.docx]

## Supplementary file 2. Intervention repository for DRC outbreaks

| **Outbreak** | **Interventions carried out** | **Dates (if known)** |
| --- | --- | --- |
| **1976, Yambuku** | Yambuku Mission Hospital closed | 30/10/1976 |
|  | Active surveillance nationwide, in Kinshasa and in the epidemic area |  |
|  | A technical note on the disease and practical advice on protection, notification and sample shipping was given to healthcare workers at the national level. Disseminated through radio and the Catholic and Protestant mission amongst others. |  |
|  | Isolation of confirmed cases in Kinshasa and quarantine of contacts for 21 days with daily temperature checks |  |
|  | Training of local active surveillance teams and provision of protective equipment |  |
|  | Involvement of the village chiefs and house-to-house surveying |  |
|  | Retrospective surveillance |  |
|  | Isolation of cases. Suspected cases were isolated in their village with no close monitoring |  |
|  | Barriers installed around villages affected to prevent entry and exit |  |
|  | Sodium hypochlorite (2%), boiling or burning were used to decontaminate or dispose of contaminated materials and waste |  |
|  | Cadavers wrapped in cloth, soaked in formalin or phenol and buried deeply |  |
|  |  | 3 |
| **1995, Kikwit** | Closure of all hospitals, health centers and laboratories in the Kikwit area (later reopened) | 04/05/1995 |
|  | Quarantine of patients in Kikwit General Hospital |  |
|  | Passive surveillance carried out by re-opened healthcare facilities |  |
|  | Active surveillance and follow-up carried out by medical students of Bandundu University |  |
|  | The establishment of a rumour registry of suspected cases and deaths |  |
|  | The use of the Kikwit Diocese radio station and church gatherings for surveillance messages |  |
|  | The distribution of protection equipment |  |
|  | The infection prevention and control training of medics and other volunteers |  |
|  | The organization of safe burials by the Red Cross |  |
|  | Leaflets, posters, banners and speeches through megaphones through the streets of Kikwit |  |
|  | Instruction of approximately 60 healthcare workers on protective equipment, disease characteristics, case definitions and management | 12/05/1995 and 14/05/1995 |
|  | Death registry established |  |
|  |  |  |
| **2007 Mweka** | Retrospective and prospective surveillance |  |
|  | Two international mobile laboratories opened, one in Mweka (PHAC) and one in Luebo (CDC) | 27/09/2007 |
|  | Organization of safe burials |  |
|  | Investigation of drinking water sources and subsequent chlorination of water |  |
|  | Disinfection of abandoned households belonging to EVD victims and all major treatment, isolation and healthcare facilities in the area |  |
|  | Implementation of protocols for adequate protective equipment, disinfection and hand hygiene amongst healthcare workers |  |
|  | Improvement of the general sanitation conditions of the SNCC hospital |  |
|  | Construction of three treatment centers, one in Kampungu, one in the Bulape Hospital and one in the Luebo General Hospital and establishment of a transport system for patients |  |
|  | Dissemination of knowledge about the disease through leaflets, radio and television education campaigns |  |
|  | Provision of free healthcare and food and medical aid for the population residing in the Kampungu area |  |
|  | Training of 185 nurses, hygiene technicians and red cross employees |  |
|  |  |  |
| **2008/9 Mweka** | Arrival of national and international support on site | 18/12/2008-25/12/2008 |
|  | Opening of first isolation center by MSF Belgium in Kaluamba | 27/12/2008 |
|  | Isolation center was transferred from Kaluamba to Kampungu with 25-bed capacity | 02/01/2009 |
|  | Three international mobile laboratories from Canada, Gabon and South Africa were built | 15/01/2009 |
|  | The establishment of a surveillance system including contact-tracing using specific forms to be completed for all cases |  |
|  | Health promotion in the community to raise awareness about the disease and how to avert infection (including sessions carried out daily in each village, meetings with authorities from many social groups (eg. priests from many religions, village chiefs, school directors), outreach through songs and messages in local radio stations, at markets and through the distribution of cassettes, response to rumours and household visits) |  |
|  | The symptomatic treatment and isolation of cases to prevent transmission in the community and improve patients’ chances of survival in an environment where appropriate protective equipment and measures were put in place. The interior of the isolation centers were fully visible from the outside to increase transparency with regard to the community. |  |
|  | Burials made safe |  |
|  | At-risk households were disinfected |  |
|  | Waste was appropriately disposed of |  |
|  | Psychological support was made available to healthcare workers, patients and their families |  |
|  |  |  |
|  |  |  |
| **2012, Isiro** | **Laboratory** |  |
|  | First laboratory was installed, a collaboration between the CDC and the Institut National de Recherche Biomédicale (INRB, Kinshasa) | 25/08/2012 |
|  | A team from the PHAC (Winnipeg) arrived and started managing the diagnostic activities together with the INRB. | 03/10/2012 |
|  | **Surveillance** |  |
|  | Retrospective surveillance (including taking blood samples, consultation of registers, family of cases, community surveys) |  |
|  | Prospective surveillance(ensuring healthcare workers use correct definitions, contact tracing and taking blood samples) |  |
|  | Creation of a harmonised database that permited real-time epidemiological analysis |  |
|  | Production of a daily bulletin |  |
|  | **Case management, funeral safety and infection control** |  |
|  | Isolation unit at the Isiro General Reference Hospital opened | 06/08/2012 |
|  | Re-organisation of treatment center at the Isiro General Reference Hospital by MSF Belgium and Spain: isolation units, handwashing stations, waste burning stations | 10/08/2012 |
|  | Personal protection equipment distributed |  |
|  | Free treatment of patients |  |
|  | Systematic (analgesic, antimalarial, antibiotics, vitamins A, B and C) and symptomatic treatments as well as three meals a day were provided |  |
|  | The safety of 37 burials was ensured: cadaver disinfected with 0.5% chlorine solution and placed in a bag before funeral and burial. When a death took place at home, the home was disinfected. Sometimes safety of burial was relayed to families and gloves were provided |  |
|  | **Water, hygiene and sanitation** |  |
|  | 220 healthcare workers, 34 technicians and 77 red cross employees were trained in disinfection techniques, preparation of chlorine solutions, secure burial and infection control in the healthcare setting. Protection and disinfection equipment was provided. |  |
|  | Disinfection of 30 households of cases and 36 healthcare centers |  |
|  | Incinerators built by a local engineer and installed in various healthcare centers |  |
|  | 13 patients were securely transferred |  |
|  | **Psychosocial support** |  |
|  | Psychological support was made available to healthcare workers, patients and their families |  |
|  | 122 families were provided kits for their reinsertion into the community (bedding, food, bucket, cup and soap) |  |
|  | Psyco-educational sessions in the community and with healthcare workers |  |
|  | Social support of families affected (eg. food) |  |
|  | 400 providers trained in psychological management of EVD cases |  |
|  | **Communication** |  |
|  | Briefing of local media, organised groups and community leaders to relay information about EVD |  |
|  | Lobbying of political and administrative authorities to gain their support. This resulted in the temporary banning of hunting, playing football and greeting in churches and the use of churches and schools to communicate messages about the disease |  |
|  | Flyers, posters, songs in Lingala and other local languages, a radio sketch, messages transmitted through the Isiro radio stations |  |
|  | 21 film projections gathering 12,450 people |  |
|  | Fast survey of the communication activities | 9/10/2012-10/10/2012 |
|  | Register of hostile reactions encountered |  |
| **2014, Boende** | **Evaluation and reinforcement of existing prospective and retrospective surveillance** |  |
|  | Training of 150 community support workers, 33 community nurses and 52 local healthcare workers |  |
|  | Harmonisation of surveillance tools |  |
|  | Creation of an a epidemiological surveillance database, a laboratory surveillance database and a treatment centre database |  |
|  | Distribution of surveillance tools |  |
|  | 21 day follow-up of the contacts of cases |  |
|  | Investigation of any alerts (ill or dead individuals) |  |
|  | Active search for new cases in the existing healthcare structures (analysis of consultation registers) and in the community (door-to-door knocking in affected villages) |  |
|  | Writing and distribution of regular surveillance reports |  |
|  | Epidemiological analysis of the outbreak dynamics as it progresses |  |
|  | Daily surveillance meetings |  |
|  | **Set-up of mobile laboratory** |  |
|  | **Case management** | Lokolia open 10/09/2014 |
|  | Two Ebola treatment centres subdivided by risk areas. One built from scratch in Lokolia (24 beds) received 40 patients and one in the General Reference Hospital of Boende (18 beds) received 12 patients. Three meals a day provided for patients and their carers |  |
|  | Home-based management of one case by a healthcare worker |  |
|  | **Communication** |  |
|  | Leaflets and posters in French and Lingala distributed to affected villages and surrounding villages (explained in households for those who spoke only Lomongo) |  |
|  | Messages and songs emitted on the local Radio Boende Bosekota |  |
|  | Briefing of community representatives (13 village chiefs, 37 healthcare workers, 91 teachers, 61 religious leaders, 3 traditional healers and 43 other community leaders) |  |
|  | Door-to-door knocking |  |
|  | Community volunteering for sensibilisation campaigns (122 support workers were trained and given bicycles, megaphones and disinfectants), transfer of patients to the treatment centers and notification of deaths and safe burials. |  |
|  | Briefing of affected households |  |
|  | Mass speeches in markets, schools and churches |  |
|  | Rapid surveys to evaluate the outcome of the sensibilisation campaigns and re-focus efforts |  |
|  | **Improvement of sanitation and hygiene** |  |
|  | Supplying protection equipment and disinfectentants to healthcare centers, two schools and the general reference hospital plus briefing on how to use these, hospital hygiene measures and how to dispose of waste |  |
|  | Distribution of soap, water treatment and disinfectants (1200 households), family Wash Kits (25 households) and hand washing buckets (400 households) in the community |  |
|  | Disinfection of 521 households, 17 schools, 16 churches and 7 healthcare centers and daily disinfection of Ebola treatment centers |  |
|  | Ensuring the safety of 18 burials |  |
|  | Evaluation of water distribution points and supply of drinking water in the meantime |  |
|  | **Psychosocial support** |  |
|  | Psychotherapy for healthcare workers, community, EVD cases and their family members (psychoeducational, supportive and community therapies) |  |
|  | Nutritional support kits distributed to patients, their families and the local population |  |
